# Venoarterial extracorporeal membrane oxygenation induces early immune alterations

**DOI:** 10.1186/s13054-020-03444-x

**Published:** 2021-01-06

**Authors:** Aurélien Frerou, Mathieu Lesouhaitier, Murielle Gregoire, Fabrice Uhel, Arnaud Gacouin, Florian Reizine, Caroline Moreau, Aurélie Loirat, Adel Maamar, Nicolas Nesseler, Amedeo Anselmi, Erwan Flecher, Jean-Philippe Verhoye, Yves Le Tulzo, Michel Cogné, Mikael Roussel, Karin Tarte, Jean-Marc Tadié

**Affiliations:** 1 Maladies Infectieuses Et Réanimation Médicale, CHU Rennes, 35033 Rennes, France; 2 INSERM, EFS Bretagne, UMR U1236, Université de Rennes 1, 35000 Rennes, France; 3 Pôle Biologie, CHU Rennes, 35033 Rennes, France; 4 Service de Bactériologie, CHU Rennes, 35033 Rennes, France; 5 Service de Cardiologie et maladies vasculaires, CHU de Rennes, 35033 Rennes, France; 6 Anesthésie-Réanimation, CHU Rennes, 35033 Rennes, France; 7 Chirurgie Cardio-Thoracique Et Vasculaire, CHU Rennes, 35033 Rennes, France

**Keywords:** VA-ECMO, Immunosuppression, MDSC, Lymphocyte exhaustion, Lymphopenia, Acquired infections

## Abstract

**Background:**

Venoarterial extracorporeal membrane oxygenation (VA-ECMO) provides heart mechanical support in critically ill patients with cardiogenic shock. Despite important progresses in the management of patients under VA-ECMO, acquired infections remain extremely frequent and increase mortality rate. Since immune dysfunctions have been described in both critically ill patients and after surgery with cardiopulmonary bypass, VA-ECMO initiation may be responsible for immune alterations that may expose patients to nosocomial infections (NI). Therefore, in this prospective study, we aimed to study immune alterations induced within the first days by VA-ECMO initiation.

**Methods:**

We studied immune alterations induced by VA-ECMO initiation using cytometry analysis to characterize immune cell changes and enzyme-linked immunosorbent assay (ELISA) to explore plasma cytokine levels. To analyze specific changes induced by VA-ECMO initiation, nine patients under VA-ECMO (VA-ECMO patients) were compared to nine patients with cardiogenic shock (control patients).

**Results:**

Baseline immune parameters were similar between the two groups. VA-ECMO was associated with a significant increase in circulating immature neutrophils with a significant decrease in C5a receptor expression. Furthermore, we found that VA-ECMO initiation was followed by lymphocyte dysfunction along with myeloid-derived suppressor cells (MDSC) expansion. ELISA analysis revealed that VA-ECMO initiation was followed by an increase in pro-inflammatory cytokines such as IL-6, IL-8 and TNF-α along with IL-10, a highly immunosuppressive cytokine.

**Conclusion:**

VA-ECMO is associated with early immune changes that may be responsible for innate and adaptive immune alterations that could confer an increased risk of infection.

## Introduction

Severe impairment of innate and adaptive immune functions has been described in critically ill state and has been associated with nosocomial infection (NI) acquisition and worst outcome [1, 2]. Identical immune dysfunctions on both monocyte and lymphocyte have been observed after cardiac surgery with cardiopulmonary bypass (CPB) and attributed to contact of blood with artificial surfaces and surgical injury, directly or indirectly impairing the function of almost all innate and adaptive immune cell subsets [3–5].

Venoarterial extracorporeal membrane oxygenation (VA-ECMO) provides mechanical support to the heart in patients with cardiogenic shock unresponsive to conventional medical therapy [6, 7]. The use of VA-ECMO has dramatically increased over the last 5 years and has been recognized as a valuable and easy to implant device, allowing myocardial recovery or bridge to cardiac transplantation or mechanical support such as left ventricular assist device (LVAD) [8, 9]. Despite important progress in the management of patients under VA-ECMO, complications and mortality rates remain extremely high [6, 10]. Notably, reactivations of quiescent viruses or infections due to opportunistic pathogens are commonly found in patients undergoing VA-ECMO [1, 11]. Although there is no consistent definition of VA-ECMO-associated infections [12], NI acquisition under VA-ECMO is particularly significant since almost two patients under VA-ECMO support out of three develop NI, with dramatic clinical consequences such as delayed cardiac transplantation or implantation of LVAD and increased mortality [11, 13, 14]. Hence, strategies to prevent or decrease NI rates in these critically ill patients will undoubtedly improve outcome. As CPB during cardiac surgery, VA-ECMO has been suspected to affect the immune system through several mechanisms such as induction of endothelial dysfunction with both activation of neutrophils, platelets and coagulation pathways [15, 16]. As a consequence, changes in pro-inflammatory interleukins (IL)-6, IL-8, tumor necrosis factor (TNF)-α and anti-inflammatory IL-10 production have been reported during VA-ECMO [17]. Although no immunological study has been performed, the complex inflammatory reaction associated with VA-ECMO initiation may favor immune dysfunctions and organ injury and therefore might increase the susceptibility to develop NI [1]. Besides functional impact on immune cells that are involved in defense against bacteria or virus reactivation such as neutrophils, dendritic cells (DC), monocytes and lymphocytes, cytokines are responsible for recruitment and expansion of myeloid suppressive cells (MDSC) [18]. MDSC have been associated with a worsened outcome and nosocomial infections in ICU patients, and various aspects of MDSC-mediated T-cell immunosuppression have been reported in other conditions where MDSC are amplified, such as cancers, obesity or after cardiac surgery with CPB [19–22]. In agreement with the suspected increase in circulating MDSC, cytokines involved in their recruitment and expansion such as IL-6 were found at higher concentration in the plasma of patients under VA-ECMO. Additionally, IL-10, one of the major factors of immune suppression mediated by MDSC, is abundant in patients under VA-ECMO [1, 19].

Therefore, the aim of our study was to prospectively study immune alterations induced within the first days by VA-ECMO initiation. Since it is challenging to discern the extent of the immunosuppression that is due solely to VA-ECMO initiation or to critical illness, patients under VA-ECMO were compared to patients admitted to ICU for cardiac failure without VA-ECMO indication.

Immune monitoring should bring evidences to the suspected underlying immunosuppression induced by VA-ECMO and responsible for secondary infections acquisition.

## Material and methods

### Patients

To analyze specific changes induced by VA-ECMO initiation, we compared patients under VA-ECMO for cardiogenic shock (VA-ECMO patients) and patients admitted to intensive care unit (ICU) for cardiogenic shock without VA-ECMO (control patients). The study protocol was approved by local ethic committee (n°16.11). Because of the observational nature of the study, a non-opposition form was provided to families and patients.

Inclusion criteria were as follows: Patients older than 18 years old hospitalized in ICU for cardiogenic shock with or without VA-ECMO. Exclusion criteria were any confirmed or suspected immunosuppressive or immune-deficient state, including HIV infection, asplenia, immunosuppressant medication within the past 6 months or implementation of immunosuppressive therapy such as chemotherapy, cyclophosphamide, high-dose corticosteroids (methylprednisolone or equivalent > 0.5 mg/kg/day) and pregnancy. Furthermore, since infection induces immune changes, patients with known or suspected infection at admission were not included in our study.

Cardiogenic shock was defined as follows: (1) systolic blood pressure < 90 mmHg for > 30 min or inotropic drugs required to achieve a blood pressure ≥ 90 mmHg; (2) pulmonary congestion or elevated left ventricular filling pressures; and (3) signs of impaired organ perfusion with at least one of the following criteria: (a) altered mental status; (b) cold, clammy skin; (c) oliguria; and (d) increased serum-lactate [23]. Since chronic cardiomyopathy has been shown to activate immune system, interval between onsets of cardiogenic shock signs and symptoms and inclusion must be < 5 days [24].

Cannulation strategy was as follows: A peripheral approach through the femoral vessels was employed. Cannulation was done using a Seldinger technique after groin incision and direct access to the femoral vein and artery. Depending on the patient’s body surface area and the vessels’ dimensions, we used inflow cannulae from 16 to 20Fr in diameter, and drainage cannulae from 18 to 32Fr in diameter (Edwards Lifesciences, Inc., Irvine, CA, USA). A reperfusion catheter was systematically introduced within the superficial femoral artery in case of peripheral VA ECMO, in order to avoid limb ischemia.

The following data were recorded: reasons for VA-ECMO initiation, gender, age, preexisting chronic kidney disease [25], preexisting chronic heart failure [23], body mass index (BMI), highest blood lactate level within 24 h following admission, SAPS II, SOFA at admission and after 24 h in ICU, duration of mechanical ventilation, duration of vasopressor treatment, length of hospital stay and outcome (alive or dead) on day 7, day 30 and day 90. The occurrence of NI was also recorded during hospital stay. NI were defined following CDC criteria as previously described [5].

Blood samples for cytometry analysis and cytokine quantification were withdrawn before VA-ECMO implantation (D0), 24 h after implantation (D1) and 4 days after implantation (D4). Blood samples were withdrawn in control group patients at ICU admission (D0), 24 h after ICU admission (D1) and 4 days after ICU admission (D4). The delay between sampling and beginning of laboratory procedures was < 1 h.

### Cytometry

Quantification of neutrophil, monocyte and dendritic cell populations was performed on whole blood by using three antibodies panels. For the neutrophil panel, whole blood was stained with CD11b PerCP5.5, CD88 (C5AR) APC, CD16 A700, CD15 Pacific Blue (Biolegend, San Diego, CA, USA), CD66b FITC, CD35 PE, CD63 PC7, CD64 APC-H7 and CD45 BV510 (Becton Dickinson, San Jose, CA, USA). Neutrophil differentiation stages were determined based on CD11b and CD16 expression. Immature neutrophils were CD16dimCD11bdim and mature neutrophils are CD16highCD11high. For the monocyte panel, whole blood was stained with CD274 FITC, CD163 PE, HLA-DR BUV395, CD64 APC-H7, CD66b BV421, CD45 BV510 (Becton Dickinson), CD14 PC7 (Beckman Coulter, Miami, FL, USA) CX3CR1 APC (Miltenyi, Bergisch Gladbach, Germany), CD16 A700, CD3 Pacific Blue and CD335 BV421 (Biolegend). Monocyte populations have been determined based on CD14 and CD16 expression. Monocytic MDSC (M-MDSC) were defined as CD14^high^ HLA-DR^low^ cells. For the dendritic cell panel, whole blood was stained with Lin FITC (Lineage 1, CD3, CD14, CD19, CD20 and CD56), HLA-DR BUV395, CD123 BV786, CD45 BV510 (Becton Dickinson), CD141 PE and CD1c APC (Miltenyi). Dendritic cell populations have been determined based on HLA-DR, CD123, CD141 and CD1c expression. After whole blood staining, erythrocytes were lysed twice with EasyLyse (Dako, Glostrup, Denmark) before washing in PBS. For granulocytic-MDSC (G-MDSC), peripheral blood mononuclear cells (PBMC) isolated after Ficoll density gradient were stained with HLA-DR PE-CF594, CD45 BV510 (Becton Dickinson), CD14 PC7 (Beckman Coulter), CD3 PE, CD16 A700, CD15 Pacific Blue (Biolegend) before washing in PBS. G-MDSC were defined as CD15^pos^ cells. Samples were assessed by flow cytometry on Fortessa X-20 (Becton Dickinson) and the results were analyzed with Kaluza 2.0 software. Expression markers were presented as mean fluorescence intensity ratio (rMFI) or ratio of mean fluorescence intensity ratio (rrMFI). rMFI was defined as the ratio between the fluorescence of stained cells and unstained cells. rrMFI was defined as the ratio between the rMFI at the studied time point and the rMFI at D0. Therefore, the rrMFI at D0 is 1.

### Proliferation assay

Peripheral blood mononuclear cell (PBMC) were labeled with carboxyfluorescein succinimidyl ester (CFSE, 200 nM; interchim, Montluçon, France) and seeded in 96-well round-bottom plates at 2.10^5^/well. Cells were cultured in RPMI 1640 supplemented with 10% human AB serum (Biowest, Nuaillé, France) and anti-CD3 and anti-CD28 monoclonal antibodies (0.6 µg/mL, Sanquin, Amsterdam, The Netherlands). After 4 days of culture, cells were harvested and labeled with CD2 PC7, CD8 APC (Beckman Coulter, Miami, FL), CD4 BUV496, CD14 BV605 (Becton Dickinson, San Jose, CA, USA) and DAPI (Sigma-Aldrich, St Louis, MO, USA). CFSE dilution was assessed on DAPI^neg^ viable T-cells by flow cytometry on Fortessa X-20 (Becton Dickinson) and the results were analyzed with ModFit LT software.

### Apoptosis assay

Whole blood was labeled with CD3 PC7, CD4 PE and CD8 APC (Becton Dickinson, San Jose, CA, USA). Erythrocytes were lysed twice with EasyLyse (Dako) before washing in PBS. Cells were resuspended in AnnexinV buffer and stained with AnnexinV FITC (Tau technologies, Kattendijke, The Netherlands) and Dapi (Sigma-Aldrich) before assessment of apoptosis by flow cytometry on Fortessa X-20 (Becton Dickinson). Data were analyzed using Kaluza 2.0 software.

### Cytokine quantification

Interleukin (IL)-6, IL-10, tumor necrosis factor (TNF)-α, granulocyte-colony stimulating factor (G-CSF), IL-8 and IL-7 were quantified in patient plasma by ELISA DuoSET (R&D system, Abingdon, UK).

### Statistical analysis

Quantitative variables are expressed as median (interquartile range, IQR) and qualitative variables as number (percentages). Continuous variables were compared using the nonparametric Mann–Whitney *U* test or Wilcoxon test for matched pairs as appropriate. Qualitative data were compared using Chi-square test or Fisher exact test when required. We considered a P value of less than 0.05 to be statistically significant. All probability values reported are two-sided. Analyses were performed with GraphPad Prism 6.2 (GraphPad Software, La Jolla, CA).

### Sample sizes

Since the effects of VA-ECMO on immune cells functions have not been previously established, there was no data available for the expected results for each experiment. However, we calculated the number of patients based on our previous results in cardiac surgery with cardiopulmonary bypass. In this study, we found that patients under VA-ECMO had a decrease in monocyte HLA-DR expression (MFI) from 7.3 to 2.8. Therefore, a total sample size of 14 patients (7 per group) was required to achieve 80% power to detect a decrease in the rate of HLA-DR expression after VA-ECMO, using a two-sided test with a type I error of 5% [5].

## Results

### Studied population

From January 2017 to January 2019, 18 patients were prospectively enrolled in the study to analyze immune changes induced by VA-ECMO initiation in the tertiary university hospital of Rennes (France). Nine patients under VA-ECMO (VA-ECMO patients) were compared to nine patients with cardiogenic shock (control patients). Baseline characteristics of the population are summarized in Table 1. Patients under VA-ECMO appeared more severely ill than control patients since duration of both mechanical ventilation and vasopressive infusion were significantly longer, although no differences were found between the two groups for SOFA or SAPS II scores at admission nor in the mortality rate. No patient received antibiotics nor immunosuppressant medication during the study period. Importantly, none of our patients presented infection at admission. Nosocomial infections were diagnosed in five VA-ECMO patients (four pneumonia and one bacteremia) and in one control (one pneumonia). The median time from ICU admission to diagnosis of nosocomial infection was 10 days [10–19].

**Table 1:** : Characteristics of the studied population

Variables	VA–ECMO *n* = 9	Control *n* = 9	*p*
Gender, *n* (%)			0.99
Male	6 (67)	7 (77)	
Female	3 (33)	2 (23)	
Age, years, median (IQR)	59 (57–63)	56 (47–68)	0.87
BMI, kg/m^2^, median (IQR)	26 (24–29)	28 (27–35)	0.19
Diabetes, *n* (%)	2 (22)	2 (22)	0.99
Cancer, *n* (%)	1 (11)	0	0.99
Chronic kidney disease, *n* (%)	0	1 (11)	0.99
Chronic heart failure, *n* (%)	2 (22)	4 (44)	0.62
Tobacco use, *n* (%)	4 (44)	1 (11)	0.29
Causes of cardiogenic shock			0.22
Acute coronary syndrome	7 (78)	3 (33)	
Acute decompensated heart failure	1 (11)	4 (44)	
Myocarditis	1 (11)	1 (11)	
Acute stress-induced cardiomyopathy	0 (0)	1 (11)	
Highest blood lactate level, median (IQR)	2.7 (2.3–4.2)	3.6 (2,6–6.8)	0.31
SAPS II, median (IQR)	45 (36–54)	41 (34–44)	0.39
SOFA baseline, median (IQR)	9 (7–13)	7 (3–9)	0.16
SOFA day 1, median (IQR)	9 (8–13)	9 (7–9)	0.32
Mechanical ventilation duration, days, median (IQR)	17 (9–21)	0 (0–14)	0.01
Vasopressor use duration, days, median (IQR)	15.5 (9.8–19.5)	6 (3–8)	0.049
Length of hospital stay, days, median (IQR)	17 (14–21)	0 (0–14)	0.001
Nosocomial infection, *n* (%)	5 (56)	1 (11)	0.13
7-day mortality, *n* (%)	1 (11)	1 (8)	0.99
30-day mortality, *n* (%)	5 (56)	4 (30)	0.99
90-day mortality, *n* (%)	5 (56)	6 (67)	0.99

VA-ECMO, venoarterial extracorporeal membrane oxygenation; BMI, Body mass index; ICU, Intensive care unit; SAPS II, Simplified acute physiology score; SOFA, Sequential organ failure assessment

### VA-ECMO initiation induced an increase in immature circulating neutrophils

**Fig. 1:**
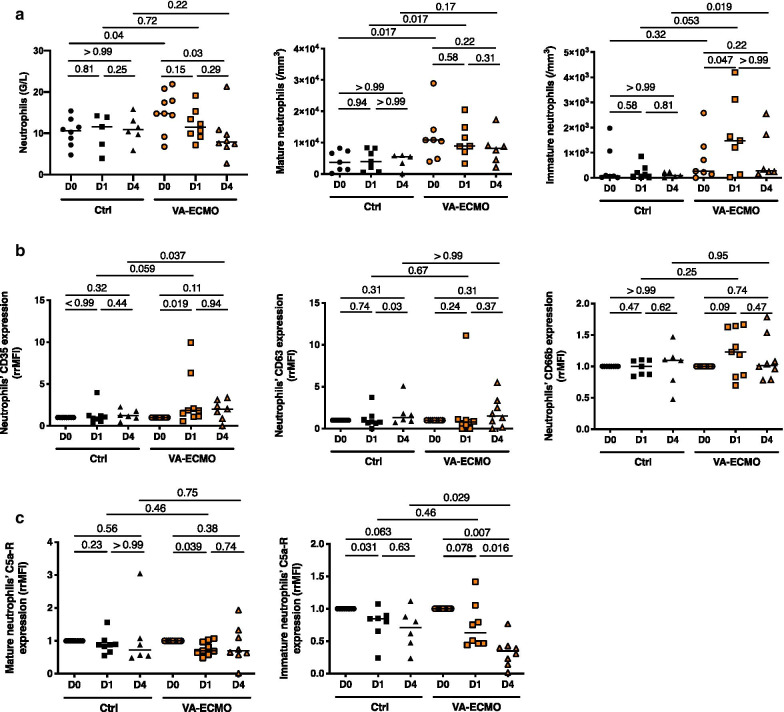
VA-ECMO induced increased in immature circulating neutrophils. **a** Total neutrophils were determined by blood count formula at admission or VA-ECMO initiation (D0), 24 h and 4 days (D1 and D4) (*n* = 9 Control (Ctrl) group, *n* = 9 VA-ECMO group). Mature and immature peripheral blood neutrophil counts were determined by flow cytometry at admission or VA-ECMO initiation (D0), 24 h and 4 days (D1 and D4) (*n* = 8 Control (Ctrl) group, *n* = 9 VA-ECMO group). **b** Surface marker of neutrophil degranulation (CD35, CD63, CD66b) was compared by flow cytometry at admission or VA-ECMO initiation (D0), 24 h and 4 days (D1 and D4) (*n* = 8 Ctrl group, *n* = 9 VA-ECMO group). Data presented as ratio of mean fluorescence intensity ratio (rrMFI), defined as the ratio of rMFI between D0 and D1 or D4. C. Expression of C5a receptor on mature and immature neutrophils compared by flow cytometry at admission or VA-ECMO initiation (D0), 24 h and 4 days (D1 and D4) (*n* = 8 Ctrl group, *n* = 9 VA-ECMO group). Data expressed as rMFI

As shown Fig. 1a, circulating neutrophils were significantly increased in VA-ECMO patients compared to controls at admission. Interestingly, while starting from similar numbers at admission, VA-ECMO patients reached a higher number of immature neutrophils at D1 and D4 compared to controls patients (D4: VA-ECMO patients (*n* = 6) 284/mm^3^ (213–1924) vs control patients (*n* = 5) 84/mm^3^ (31–228), *p* = 0.019). Although expression of the degranulation markers CD35, CD63 and CD66b remained unchanged after VA-ECMO initiation and similar to controls (Fig. 1b), we found that the C5a receptor, which has been associated with ability of neutrophils to kill cocci gram positive bacteria [26], significantly decreased within 24 h following VA-ECMO initiation, in both mature and immature neutrophils (Fig. 1c).

### VA-ECMO initiation induced changes in antigen presenting cell phenotype

**Fig. 2:**
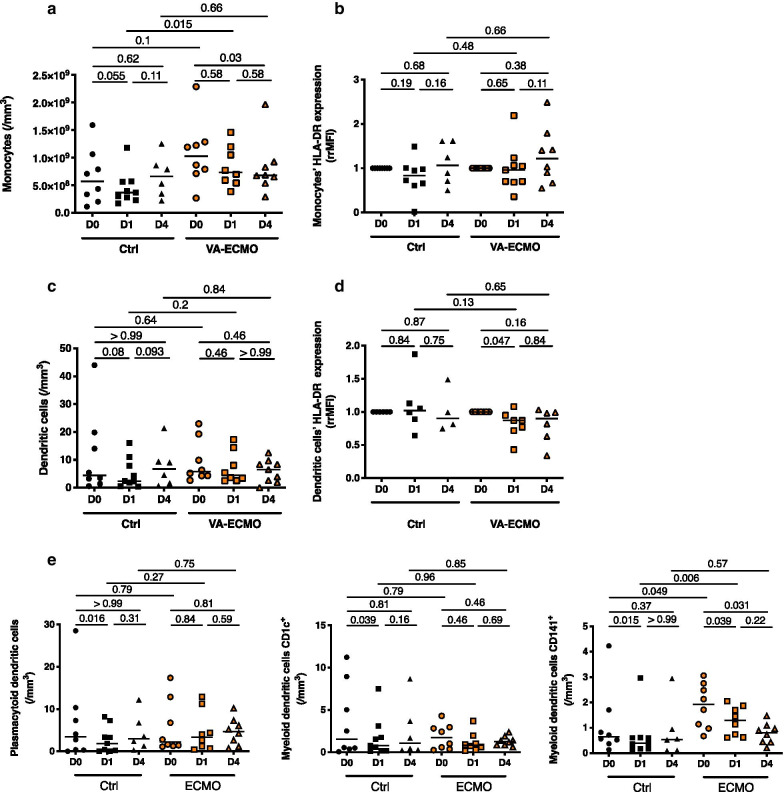
VA-ECMO induced changes in antigen presenting cell phenotype. **a** Peripheral monocyte counts were determined by flow cytometry at admission or VA-ECMO initiation (D0), 24 h and 4 days (D1 and D4) (*n* = 8 Control (Ctrl) group, *n* = 9 VA-ECMO group). **b** Monocyte HLA-DR expression was measured by flow cytometry at admission or VA-ECMO initiation (D0), 24 h and 4 days (D1 and D4) (*n* = 8 Control (Ctrl) group, *n* = 9 VA-ECMO group). HLA-DR expression has been represented as ratio of mean fluorescence intensity ratio (rrMFI) defined as the ratio of rMFI between D0 and D1 or D4. **c** Peripheral dendritic cell (DC) counts were determined by flow cytometry at admission or VA-ECMO initiation (D0), 24 h and 4 days (D1 and D4) (*n* = 8 Control (Ctrl) group, *n* = 9 VA-ECMO group). **d** Dendritic cells HLA-DR expression was measured by flow cytometry at admission or VA-ECMO initiation (D0), 24 h and 4 days (D1 and D4) (*n* = 8 Control (Ctrl) group, *n* = 9 VA-ECMO group). HLA-DR expression has been represented as ratio of mean fluorescence intensity ratio (rrMFI) defined as the ratio of rMFI between D0 and D1 or D4. (*n* = 8 Control (Ctrl) group, *n* = 9 ECMO group). E. Peripheral dendritic cell (DC) subsets counts (plasmacytoid DC (pDC) and myeloid DC (mDC CD1cpos and mDC CD141pos)) were determined by flow cytometry at admission or VA-ECMO initiation (D0), 24 h and 4 days (D1 and D4) (*n* = 8 Control (Ctrl) group, *n* = 9 VA-ECMO group)

Monocytes and dendritic cells (DC) are important antigen-presenting cells and play a key role in immune response by regulating the innate and adaptive immunity. The number of DCs, the differentiation of monocytes into DC and the levels of surface molecules associated with the function of DC undergo important changes in critically ill patients [27, 28]. VA-ECMO did not induce changes in the number of circulating monocytes number nor in their expression of HLA-DR (Fig. 2a, b). On the contrary for DCs, although their number was not affected, HLA-DR expression decreased after VA-ECMO initiation (DCs HLA-DR expression (rrMFI) at D0 1 (1–1) (*n* = 7) vs 0.87 (0.72–0.95) (*n* = 7) at D1, *p* = 0.047) (Fig. 2c, d). Furthermore, when analyzing DC subsets, we found that the number of circulating mDC CD141^pos^, decreased significantly after VA-ECMO initiation and persisted at D4, although numbers of the two other circulating DC subsets plasmacytoid DC (pDC) and myeloid DC (mDC CD1c^pos^) remained unchanged after VA-ECMO initiation (Fig. 2e).

### VA-ECMO initiation induced MDSC expansion and T-cell dysfunction

**Fig. 3:**
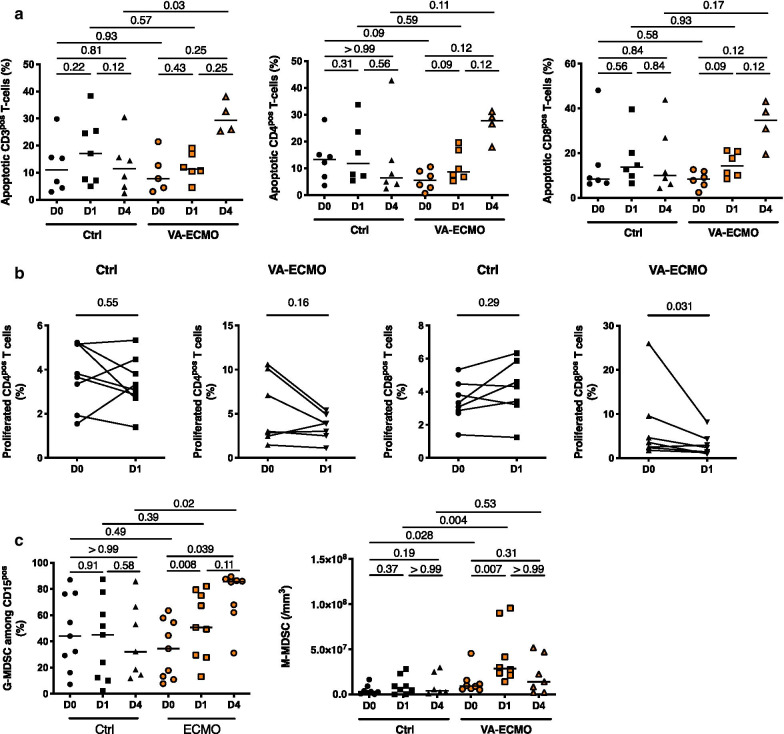
VA-ECMO induced MDSC expansion and T-cell dysfunction. **a** Total lymphocytes were determined by blood count formula. **b** Proportion of apoptotic T-cells and their subpopulations, CD4^pos^ and CD8^pos^, were determined at admission or VA-ECMO initiation (D0), 24 h and 4 days (D1 and D4) (*n* = 6 Control (Ctrl) group, *n* = 6 VA-ECMO group) by flow cytometry with an annexinV binding assay (*n* = 6). Results expressed as percentage of AnnexinV^pos^ Dapi^neg^ cells. **c** Fresh PBMC obtained at admission or VA-ECMO initiation (D0) and 24 h after (D1) were stimulated with anti-CD3/anti-CD28 monoclonal antibodies after CFSE labelling. The proportion of CD4^pos^ (two left graphics) and CD8^pos^ (two right graphics) proliferated T cells were determined by flow cytometry (*n* = 9 Ctrl group, *n* = 7 ECMO group). **d** Percentage of granulocytic (G)-MDSC among PBMCs was determined by flow cytometry at admission or VA-ECMO initiation (D0), 24 h and 4 days (D1 and D4) (*n* = 8 Control (Ctrl) group, *n* = 9 VA-ECMO group). Peripheral monocytic (M)-MDSC counts were determined by flow cytometry at admission or VA-ECMO initiation (D0), 24 h and 4 days (D1 and D4) (*n* = 8 Control (Ctrl) group, *n* = 9 VA-ECMO group)

As shown in Fig. 3a, late T-cell apoptosis which has been associated with mortality and nosocomial infection acquisition was enhanced in VA-ECMO patients compared to control group (Apoptotic CD3^pos^ T-Cells at D4 11.49% (4.14–19.32) in controls (*n* = 6) vs 29.26% (25.43–36.66) in VA-ECMO patients (*n* = 4) *p* = 0.003) [29].

Moreover, T-cell proliferation was decreased within 24 h following VA-ECMO initiation although no changes were observed in control patients (Fig. 3b). Along these T-cells dysfunctions, we observed the expansion of myeloid-derived suppressor cells (MDSC). MDSC consist of two major subsets of granulocytic (G-MDSC) and monocytic cells (M-MDSC) and have been described as strongly immunosuppressive [30]. Initiation of VA-ECMO was associated with expansion of MDSC, and we found that both circulating G-MDSC and M-MDSC were significantly expanded after VA-ECMO initiation but with a different kinetic (Fig. 3c). The number of M-MDSC was markedly increased in VA-ECMO patients (*n* = 8) at D1 (28.5 × 10^6^/mm^3^ (21.7 × 10^6^–77.8 × 10^6^) compared to D0 (9.27 × 10^6^/mm^3^ (5.92 × 10^6^–14.5 × 10^6^), *p* = 0.007). Of note, we found that patients with nosocomial infection had a higher M-MDSC count at day 1 compared to patients without nosocomial infections (6.5 × 10^7^/mm^3^ (3.2 × 10^7^–9.0 × 10^7^) vs. 1.7 × 10^7^/mm^3^ (6.8 × 10^7^–2.4 × 10^7^), respectively, *p* = 0.002).

### Cytokine analysis

**Fig. 4:**
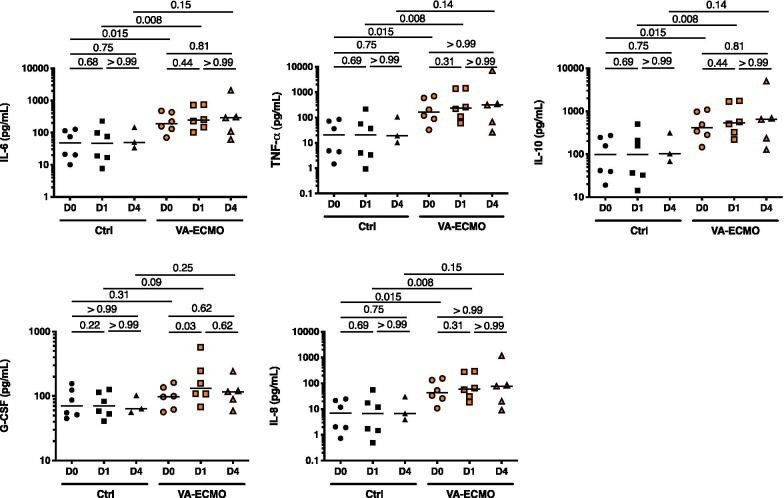
Cytokine analysis in patients under VA-ECMO and control patients. Quantification of interleukine (IL)-6, tumor necrosis factor (TNF)-α, IL-10, granulocyte-colony stimulating factor (G-CSF) and IL-8 by ELISA in plasma from patients at admission or VA-ECMO initiation (D0), 24 h and 4 days (D1 and D4) (*n* = 6 Control (Ctrl) group, *n* = 6 VA-ECMO group)

Cytokine analysis in patients under VA-ECMO revealed a preexisting inflammatory response with significantly higher plasma levels of pro-inflammatory cytokines such as IL-6, IL-8 and TNF-α compared to control patients at admission. Plasma levels of these pro-inflammatory cytokines remained increased after VA-ECMO initiation. Furthermore, we found higher levels of IL-10 in patients under VA-ECMO compared to controls. Noteworthy, plasmatic levels of IL-7 were not analyzable since most of the data were below the limit of detection, and G-CSF was increased within 24 h after VA-ECMO (Fig. 4).

## Discussion

Our study reveals that VA-ECMO initiation is associated with immune changes responsible for an immunosuppression which may explain the high incidence of acquired infections observed in these patients. These changes occurred within 24 h following VA-ECMO initiation with major modifications in crucial immune functions that have been associated with nosocomial infections. Our results confirm the suspected immune dysfunction that comes along with interleukin changes observed in previous studies.

As immune changes observed in patients undergoing cardiac surgery with CPB, VA-ECMO was suspected to strongly weaken the adaptive immune system [3, 20]. However, our study brings evidences that VA-ECMO initiation also affected innate cells. First, we found an increased proportion of immature neutrophils within 24 h following VA-ECMO initiation, along with an increased G-CSF plasma level. Immature neutrophils could display immunosuppressive properties with impaired phagocytosis and bactericidal activities, thus increasing the risk of secondary infections [31, 32]. They arise under stimulation by G-CSF, a key granulopoietic cytokine, the secretion of which is increased after VA-ECMO initiation [33]. As observed in critically ill patients, we found that neutrophils of patients under VA-ECMO underwent modification that could affect their function and their ability to effectively kill invading organisms such as decreased C5a receptor expression, a key receptor for phagocytosis of *Staphylococcus aureus,* a bacteria commonly responsible for acquired infection [34]. Since neutrophil-mediated killing is the most important mechanism against *Staphylococcus aureus* infection, these modifications may have dramatic consequences [1, 26]. Our findings highlight the specificity of changes induced by VA-ECMO compared to those observed in patients undergoing cardiac surgery with CPB as it has been found that cardiac surgery with CPB significantly increased the number of circulating neutrophils with enhanced ability to kill bacteria [35]. Noteworthy, we observed no changes in monocyte expression of HLA-DR after VA-ECMO initiation. This last result was unexpected because early and significant changes in monocyte expression of HLA-DR are usually reported in patients undergoing cardiac surgery with CPB, although it could be related to a very low monocyte expression of HLA-DR in severely ill VA-ECMO patients at admission which is not usually observed in patients before undergoing cardiac surgery with CPB [5, 36, 37]. However, we found that VA-ECMO induced a transient decrease in the HLA-DR expression on DC along to a significant decrease in the number of circulating mDC CD141^pos^ subset, the main human DC subset involved in infection control [38]. Such differences in immune changes observed could be related to the duration of inflammatory stimulation. The immune response to strong stimulation from pathogens or surgical procedure with CPB consists into a rapid mobilization of short duration of monocytes or neutrophils with enhance capacity in phagocytosis and respiratory burst [27, 39]. During unresolved inflammation, neutrophils and monocytes arise with an immature phenotype along with markers of immunosuppression [40].

Most importantly, we demonstrated that VA-ECMO induced MDSC expansion and T-cell dysfunction. MDSC levels are increased in critically ill patients and after cardiac surgery [5, 18, 30]. These cells can impair the adaptive immune system by suppressing CD4^pos^ and CD8^pos^ T-cell activation and function and can promote their apoptosis, altogether favoring infection and mortality in septic patients [30, 41, 42]. MDSC consist of two large groups of cells termed G-MDSC, which are phenotypically and morphologically similar to neutrophils, and M-MDSC, which are more suppressive than G-MDSC and phenotypically and morphologically similar to monocytes [40, 43]. We found that both G-MDSC and M-MDSC were significantly increased in patients with VA-ECMO although we did not find MDSC expansion in patients with cardiogenic shock treated medically. Both subsets are released from the bone marrow after stimulation with various inflammatory/infectious signals such as G-CSF and IL-6 which were more increased after VA-ECMO initiation compared to patients with cardiogenic shock without ECMO [18]. Although MDSC are implicated in the suppression of different cells of the immune system, the main targets of MDSC are T-cells [19]. Along these lines, patients under VA-ECMO presented T-cells dysfunctions with increased T-cells apoptosis and decreased ability of T-cells to proliferate. Lymphocyte dysfunction has been initially described in chronic infection and has been related to late death in septic patients and increased risk of nosocomial infection [28]. Apoptotic lymphocytes are characterized by immunosuppressive properties through liberation of Damage Associated Molecular Patterns (DAMPS) [29, 44, 45] and MDSC induce T-cells dysfunctions through various mechanisms such as depleting the extracellular milieu in amino-acids which induce cellular metabolism dysfunction [46, 47].

Lastly, IL-7, which promotes T-cells viability and functionality and improves survival in critically ill patients [48, 49], was mainly undetectable in the plasma from VA-ECMO patients, and we found higher levels of IL-10 in patients under VA-ECMO compared to cardiogenic shock patients. IL-10 is an interleukin known to decrease the ability of DC and macrophages to stimulate the proliferation of T-cells, and it has been shown that addition of anti-IL-10 almost completely blocked the anti-proliferative effects of MDSCs on T cells [50–52].

This study has several limitations that have to be acknowledged. To study specific effects of VA-ECMO initiation in cardiogenic shock, we decided to include, as controls, ICU patients with cardiogenic shock treated medically. Although closely related to VA-ECMO patients, this control group featured a less severe cardiac status responsive to medical treatment*.* While these groups did not differ in terms of starting immune status, marked immune changes appeared within 24 h after VA-ECMO initiation. The strict inclusion criteria and the methodology used meant that only few patients could be included. As a consequence, only 18 patients have been included over a two years period. Small size of both groups undoubtedly limits the value of our results. For instance, the trend for differences in cytokine levels could indicate that the study is under-powered. Of note, no practice changes have been observed during the study period. The VA ECMO patient population and the control patient population have important differences in rate of mechanical ventilation and median days of vasopressor use, and the differences observed in our study could also be related to the severity of shock at admission. For instance, both of these differences may have important implications for MDSC expansion [53]. The function of monocytes has not been studied through functional testing such as cytokine production under stimulation. We only described the number of monocytes, and the levels of surface molecules which could be associated with their function. Since it has been shown that monocytes from critically ill patients (septic and non-septic patients) produce lower amount of cytokine and that survival patients recovered their capacity to produce normal amounts of cytokines upon stimulation, performing these experiments would have been of great value [54]. Lastly, we acknowledge that our study does not provide an exhaustive analyze in immune changes, and we did not conduct *in-vitro* experiment to demonstrate the role of MDSC in T cell dysfunction. However, our findings allow us to suggest that VA-ECMO induces profound changes in immune cells that may expose patients to develop infections.

## Conclusions

In conclusion, we found that VA-ECMO induced early immune changes identical to those associated with increased risk of infection acquisition and mortality in critically ill patients. A better knowledge of mechanisms involved would allow therapeutic interventions to decrease VA-ECMO associated infections and decrease mortality.

## Data Availability

The datasets used and/or analyzed during the current study are available from the corresponding author on reasonable request.
